# Chai-Qin-Cheng-Qi Decoction and Carbachol Improve Intestinal Motility by Regulating Protein Kinase C-Mediated Ca^2+^ Release in Colonic Smooth Muscle Cells in Rats with Acute Necrotising Pancreatitis

**DOI:** 10.1155/2017/5864945

**Published:** 2017-04-26

**Authors:** Chen-Long Zhang, Zi-Qi Lin, Rui-Jie Luo, Xiao-Xin Zhang, Jia Guo, Wei Wu, Na Shi, Li-Hui Deng, Wei-Wei Chen, Xiao-Ying Zhang, Shameena Bharucha, Wei Huang, Robert Sutton, John A. Windsor, Ping Xue, Qing Xia

**Affiliations:** ^1^Department of Integrated Traditional Chinese and Western Medicine, Sichuan Provincial Pancreatitis Centre, West China Hospital, Sichuan University, Chengdu, China; ^2^NIHR Liverpool Pancreas Biomedical Research Unit, Royal Liverpool University Hospital NHS Trust, University of Liverpool, Liverpool, UK; ^3^Department of Surgery, University of Auckland, Auckland, New Zealand

## Abstract

Chai-Qin-Cheng-Qi decoction (CQCQD) improves intestinal motility in acute pancreatitis (AP), but the mechanism(s) require elucidation. We investigated the effects of CQCQD and carbachol, a prokinetic agent, on colonic smooth muscle cells (SMCs) in L-arginine-induced necrotising AP model in rats. In treatment groups, intragastric CQCQD (20 g/kg, 2 hourly × 3 doses) or intraperitoneal carbachol (60 *μ*g/kg) was given 24 hours after induction of AP. Both CQCQD and carbachol decreased the severity of pancreatic and colonic histopathology (all *P* < 0.05). Both CQCQD and carbachol reduced serum intestinal fatty acid binding protein, vasoactive intestinal peptide, and substance P and increased motility levels. CQCQD upregulated SMC phospholipase C-beta 1 (PLC-*β*1) mRNA and PLC protein (both *P* < 0.05), while both treatments upregulated protein kinase C-alpha (PKC-*α*) mRNA and PKC protein and downregulated adenylate cyclase (AC) mRNA and protein compared with no treatment (all *P* < 0.05). Neither treatment significantly altered L-arginine-induced PKC-*β*1 and PKC-*ε* mRNA reduction. Both treatments significantly increased fluorescence intensity of SMC intracellular calcium concentration [Ca^2+^]_i_ (3563.5 and 3046.9 versus 1086.9, both *P* < 0.01). These data suggest CQCQD and carbachol improve intestinal motility in AP by increasing [Ca^2+^]_i_ in colonic SMCs via upregulating PLC, PKC and downregulating AC.

## 1. Introduction

Acute pancreatitis (AP) represents one of the most common digestive diseases requiring emergency hospital admission with an increasing incidence globally [1]. Persistent organ failure is the most important determinant of mortality in AP [2–4]. Occurring during the first week or later in the disease course, it is associated with a mortality rate of around 30% [4], which increases to 50% with persistent multiple organ failure [4, 5]. The recommended scoring systems for organ failure [6, 7] include the respiratory, cardiovascular, and renal systems but do not include the gastrointestinal system. Gut dysfunction is common in AP, and ileus is the most frequent complication (68%) and it is more common than respiratory failure (37%) [8]. Further, aspects of the management of AP can contribute to gut dysfunction, including nonselective inotropes (increased splanchnic vasoconstriction), enteral nutrition (increased metabolic demand), antibiotics (causing dysbiosis), and aggressive fluid resuscitation (oedema, reperfusion injury, and abdominal compartment syndrome) [9–11]. Recognition that the management of AP can contribute to gut dysfunction has led to the concept of “gut rousing,” where attention is directed towards maintaining gut function [12]. While there is no established marker of gut dysfunction [13], it is well documented that severe AP is associated with impaired gut microcirculation [14], motility [15], mucosa [16], barrier function [17], development of endotoxaemia [18], and altered gut-lymph components [19, 20]. Despite many studies seeking to understand the basis of gut dysfunction in AP, few have examined the intracellular signal transduction pathways in the intestinal smooth muscle cells (SMCs).

In gastrointestinal SMCs, cholinergic neurotransmitters such as acetylcholine activate muscarinic M3 or M2 receptors that signal through G proteins to regulate contractile force [21]. The M3 receptors bind to Gq proteins and regulate cellular Ca^2+^ levels via activation of phospholipase C-beta (PLC-*β*) and protein kinase C (PKC) signalling pathways [21]. Activated PKC also contributes to decreased activity of adenylate cyclase (AC) which is one of the earliest and best-described effects of M2 receptor activation in SMCs and is suppressed through activated Gi proteins via M2 receptors [21]. Of the 12 human isoforms of PKC mRNA [22] three are for Ca^2+^-dependent PKC including *α*, *β*1 and the novel Ca^2+^-dependent isoform *ε*, which is found in intestinal SMCs [23].

Two treatments have been shown to positively impact gut function, but little is known of their mechanism of action. Carbachol, a muscarinic cholinergic agonist primarily activating M2 and M3 receptors in SMCs, has been shown to reduce cytokine release and promote gut function in experimental burns shock [24] and sepsis [25]. These effects may be due to the fact that carbachol induces contractions in intestinal SMCs both in vitro and in vivo [26] by increasing the cytosolic Ca^2+^ concentration ([Ca^2+^]_i_) [27]. A second treatment is rhubarb* (Radix et Rhizoma Rhei)* and its Chinese herbal formula Chai-Qin-Cheng-Qi (CQCQD), which have been shown by recent systematic review [28] and meta-analyses [29, 30] to reduce incidence of organ failure, need for surgical intervention, and mortality in AP. Recent clinical trials have also shown that rhubarb [31] and CQCQD derivatives [32–35] reduce intra-abdominal pressure, shorten the duration of ileus, and improve clinical outcomes. The mechanism by which this treatment effect occurs is unknown but could be through Ca^2+^-dependent intracellular signalling pathways in intestinal SMCs [21].

Repeated injections of high doses of L-arginine induce AP in rodents with well-defined, gradually progressive pancreatic necrosis and associated lung injury [36]. Therefore, they are advantageous for addressing the molecular mechanisms and regenerative processes in necrotising AP. In this study, we sought to use L-arginine-induced necrotising AP in rats and investigate whether CQCQD and carbachol act through PKC-mediated Ca^2+^ signalling pathways in colonic SMCs, thus improving intestinal motility.

## 2. Materials and Methods 

### 2.1. Animals

Adult male Sprague-Dawley rats (200–300 g) were purchased from the Experimental Animal Centre of West China Centre of Medical Sciences of Sichuan University (Chengdu, China). Animals were housed at 23 ± 2°C with a 12 h light-dark cycle and were allowed free access to water and standard laboratory chow. All animal studies were performed according to the Guide for the Care and Use of Laboratory Animals of the National Institutes of Health. The protocol was approved by the Committee on the Ethics of Animal Experiments of the Sichuan University.

### 2.2. CQCQD Preparation and Reagents

The Chinese medicinal herbs in CQCQD were purchased from the West China Hospital of Sichuan University (Chengdu, China). The herbs included the following:* Bupleuri Radix* (Bupleurum) 15 g*, Scutellariae Radix *(Scutellaria) 15 g*, Radix et Rhizoma *(Rhubarb) 20 g*, Natrii Sulfas* (Mirabilite) 20 g*, Magnoliae Officinalis Cortex* (Magnolia officinalis) 15 g*, Aurantii Fructus Immaturus* (Immature Bitter Orange) 15 g*, Artemisiae Scopariae Herba* (Capillary Wormwood Herb) 15 g, and* Gardeniae Fructus* (Gardenia fruit) 20 g. These herbs were first made into 200 mL decoction and then were lyophilised into powder. The lyophilised powder of CQCQD was prepared at a concentration of 2 g/mL of crude herbs before administration to animals. Unless otherwise stated, all other reagents used in this study were of highest grade from Sigma-Aldrich (Shanghai, China). L-Arginine-HCl was freshly prepared before each experiment by dissolving in normal saline, and its pH was adjusted to 7.2 with NaOH. The concentration of L-arginine stock was 20% (w/v).

### 2.3. AP Model and Treatments

Rats were randomly allocated to 4 groups: (1) control group: intraperitoneal normal saline (2.5 g/kg, 2 injections, 1 hour apart); (2) untreated AP group: intraperitoneal L-arginine (2.5 g/kg, 2 injections, 1 hour apart) [37]; (3) CQCQD treated group: L-arginine and intragastric CQCQD (20 g/kg, 2 hourly × 3 doses); or (4) carbachol treated group: L-arginine and intraperitoneal carbachol (60 *μ*g/kg, single injection). The effects of CQCQD (treatment protocol) were also assessed in rats receiving only intraperitoneal saline injections. For untreated AP group, rats were humanely sacrificed at designated time points to investigate the changes of L-arginine-induced pancreatic and colonic damage. Both treatments began 24 hours after AP induction (mimicking therapeutic rather than preventive) and all rats were sacrificed at 30 hours after AP induction for assessment of disease severity and isolation of colonic SMCs.

### 2.4. Assessment of Pancreatic and Colonic Histopathology

After the removal of pancreatic and colonic tissues, the sections of samples were fixed in 10% neutral buffer formaldehyde, embedded with paraffin wax, cut into slices, stained with hematoxylin and eosin (H&E), and then observed under light microscopy. For each pathological section, 10 visual fields under a high-power microscope (×200) were randomly selected and scored by a pathologist. The mean score of the 10 visual fields per section was calculated as the histopathological score. For pancreatic histopathology, oedema, inflammation and perivascular infiltrate, haemorrhage and fat necrosis, and acinar necrosis were evaluated and scored according to the standard criteria [38]. For colonic histopathology, a previous protocol [39] was used with modification: the overall histopathology score was the sum of severity of inflammation (none, slight, moderate, and severe), extension of inflammation (none, mucosa, mucosa and submucosa, and transmural), and Crypt damage (none, basal 1/3 damaged, basal 2/3 damaged, and only surface epithelium intact).

### 2.5. Measurement of Indirect Intestinal Motility Biomarker Levels

After sacrifice of the rats, serum was collected by centrifuging the blood at 1500*g* for 10 min. Serum intestinal fatty acid binding protein (iFABP), vasoactive intestinal peptide (VIP), motilin (MTL), and substance P (SP) levels were measured by Enzyme Linked Immunosorbent Assay (ELISA) kits (Cusabio Biotech Co. Ltd., Wuhan, China) according to manufacturer's instructions.

### 2.6. Isolation of Colonic SMCs

Isolation of colonic SMCs followed a previously established procedure with minor modifications [40]. The abdominal cavity of rats was opened along the ventral line and approximately 10 cm of colon was obtained starting at 2 cm from the anus immediately after sacrifice. The colon was immediately soaked in phosphate-buffered saline (PBS) containing 100 U/mL penicillin/streptavidin. The colon was repeatedly washed with PBS buffer to remove blood, fat, and faeces. The tissue was minced into small fragments and digested in Dulbecco's modified Eagle's medium (DMEM) containing 1 g/L collagenase II and 0.01% soybean trypsin inhibitor solution at 31°C in a shaking water bath for 2 × 20 min (120 cycles/min). A stop solution of DMEM containing 2 g/L bovine serum albumin (BSA) was added to stop the digestion. The cells were resuspended with a plastic pipette, filtered through a nylon mesh (100 meshes), and layered into DMEM. The SMCs were centrifuged three times at 800 rpm/min for 4 min. The cells were resuspended before being used in fresh DMEM medium containing 2 g/L BSA. Confirmation that the suspended cells were SMCs was done by *α*-smooth muscle actin (*α*-SMA) staining.

### 2.7. Quantitative Real-Time PCR (RT-PCR)

The total RNA of SMCs was extracted using TRIzol reagent (Invitrogen, San Diego, USA) after collection. Following centrifugation (1,2000 rpm × 10 min) at 4°C, supernatant was transferred to ribonuclease- (RNase-) free Eppendorfs. After placement at room temperature for 5 min, 0.2 mL of chloroform was added to each sample, followed by 15 s vortex and 2 min room temperature incubation. Following centrifugation (1,2000 rpm × 15 min) at 4°C, the aqueous phase was transferred to Eppendorfs; then 0.5 mL isopropanol was further added, mixed, and sedimented for 10 mins. After centrifugation (1,2000 rpm × 10 min) at 4°C, the supernatant was discarded and the pellet was resuspended in 1 mL 75% ethanol. The sample was further centrifuged (1,2000 rpm × 5 min) at 4°C, supernatant discarded, RNA pellet air-dried, dissolved in 20 *μ*L RNase-free water, and stored at −80°C before agarose gel electrophoresis. For agarose gel electrophoresis, 0.4 g agarose (Biowest, USA) in 40 mL electrophoresis buffer (1x Tris-Borate-EDTA) was heated until dissolved; then the mixture was cooled to 60°C and 2.5 *μ*L of 10 mg/mL ethidium bromide (EB) was further added to stain the RNA. Then the EB containing 1% agarose was loaded into gel cassette and placed at room temperature for 30 min for solidification before electrophoresis. RNA sample or ladder (each 5 *μ*L) with 1 *μ*L 6x DNA loading buffer (TransGen Biotech, Beijing, China) was loaded and run at 120 V for 30 min. The integrity of the total RNA was assessed using an ultraviolet spectrophotometer (Thermo Fisher, Waltham, USA). Intact total RNA had clear 28S and 18S rRNA bands and their ratio was approximately 2 : 1. Partially (smeared appearance of the bands) or completed degraded samples (a very low molecular weight smear) were not used for the following steps. The total RNA was reverse-transcribed using hexanucleotide random primers with superscript II RNase H-reverse transcriptase (Life Technologies, Carlsbad, USA). The cDNA was amplified as a template using the specific primers (all of them were from Shanghai Biotechnology Co. Ltd.; Table 1) for subsequent RT-PCR by Taq DNA polymerase in a Perkin Elmer Cetus DNA thermocycler (Funglyn Biotech Inc., Markham, Canada). The reaction system of RT-PCR included 5 *μ*L cDNA room temperature product, 3 *μ*L 10x PCR buffer, 3 *μ*L MgCl_2_ of 25 mmol/L, 0.36 *μ*L dNTP of 25 mmol/L, 1 *μ*L upstream primer of 10 *μ*mol/L, 1 *μ*L downstream primer of 10 *μ*mol/L, 0.6 *μ*L probe of 10 *μ*mol/L, 0.3 *μ*L Taq polymerase of 5 U/*μ*L, and 15.74 *μ*L deionised double-distilled water. The RT-PCR cycling conditions were constant as follows: initial denaturation at 94°C for 2 min, and then followed by 94°C for 20 sec, with each followed by 52°C (PLC-*β*1), 54°C (PKC-*α*), 52°C (PKC-*β*1), 52°C (PKC-*ε*), 58°C (AC), and 54°C (*β*-actin) for 30 sec by 45 cycles and then 60°C for 40 sec.

### 2.8. Western-Blotting Analysis

The SMCs in each group were solicited using the Sonics Vibracell sonicator with a 0.4 mm diameter probe. Proteins in each sample were determined by a Bradford assay (Bio-Rad, Hercules, USA). The samples were denatured at 95°C for 3 min in 1x Nu Page sodium dodecyl sulphate sample buffer (Life Technologies, Carlsbad, USA). Equal amounts of protein were loaded, electrophoresed, and transferred to Immobilon-P membranes (Merch Millipore, Darmstadt, Germany). The membranes were blocked overnight in 5% BSA, followed by 3 h incubation in primary antibodies (0.5 *μ*g/mL) at room temperature. Then the membranes were washed and incubated with secondary antibodies for 1 hour. Antibodies for phospholipase C-beta 1 (ab77743, 1 : 300), protein kinase C (ab23511, 1 : 300), adenylate cyclase (ab124241, 1 : 300), and *β*-tubulin (ab15568, 1 : 1000) were from Abcam (Cambridge, UK). The protein of *β*-tubulin was used as internal housekeeping protein. Immunoreactivity was detected using a chemiluminescence's system and quantified by using Image J software (Bio-Rad, Hercules, USA).

### 2.9. Measurement of [Ca^2+^]_i_

To remove the DMEM medium, the SMCs were washed with Hank's Balanced Salt Solution (HBSS) 2 times. The cells were loaded with Fluo 3-AM (5 *μ*mol/L; excitation, 488 nm; emission 505–550 nm) in a cell incubator for 30 min at 37°C and then were washed 2 times with HBSS. The fluorescence of the Fluo 3-AM loaded cells was recorded using a Leica FV1000 laser scanning confocal microscope (Olympus, Tokyo, Japan). Twenty different cells in each visual field were randomly selected. The relative fluorescence was analysed using fluorescence quantitative analysis software and the intracellular calcium fluorescence intensity (FI) was presented as intracellular [Ca^2+^]_i_.

### 2.10. Statistical Analysis

Data are expressed as means with standard errors of means (SEM). In all figures, vertical bars denote means ± SEM values. Statistical evaluation of the data was accomplished using a Student's *t*-test where data were normally distributed or otherwise a Mann–Whitney* U* test was adopted. The Relative Expression Software Tool was employed for statistical analysis of gene expression. Statistical analysis was performed using the statistical software package Origin 8.5 (OriginLab, Northampton, MA, USA) and a value of *P* < 0.05 was considered to be significant.

## 3. Results

### 3.1. L-Arginine Induces Necrotising AP, Colonic Damage, Intestinal Dysmotility, and Aberrant SMC Ca^2+^ Signalling

**Figure 1:**
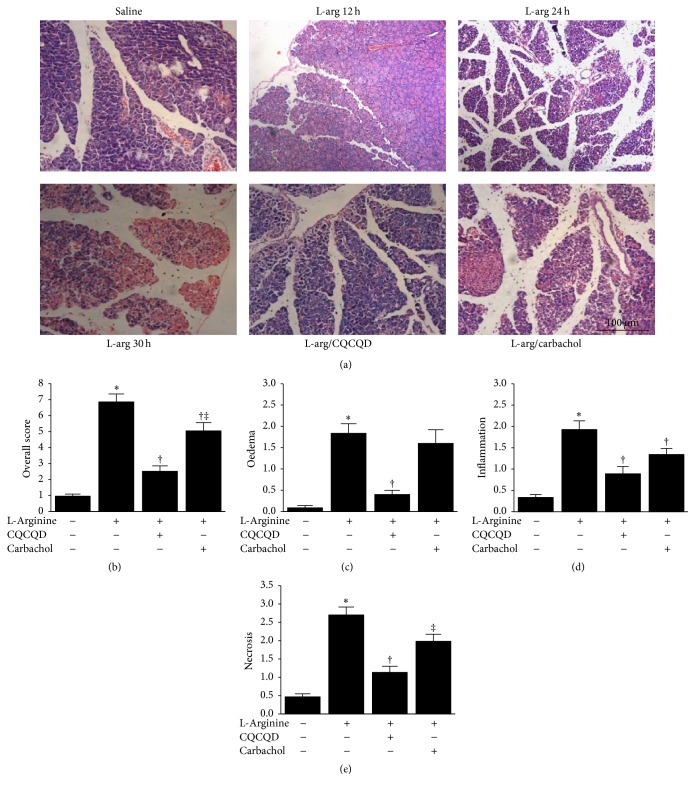
Effects of CQCQD and carbachol on pancreatic histopathology. (a) Representative H&E sections of the head of the pancreas (×200). (b) Overall histopathological score and breakdown scores: (c) oedema, (d) inflammation, and (e) necrosis. Haemorrhage score is not included. ^*∗*^*P* < 0.05 versus control group; ^†^*P* < 0.05 versus L-arginine group; ^‡^*P* < 0.05 versus CQCQD treated group. Values are means ± SEM of 5–11 animals per group.

Representative H&E pancreatic sections are shown in Figure 1(a). Saline injections did not cause any discernible histopathological changes of the pancreas. At 12 hours after L-arginine administration, only mild oedema and inflammatory cell infiltration were observed; at 24 hours, considerable oedema, inflammatory cell infiltrates, and patchy acinar cell necrosis were noticed; at 30 hours, significant isolation of pancreatic lobes, marked oedema, inflammatory cell infiltration, haemorrhage and acinar cell necrosis, and all typical features of necrotising AP were detected. These pancreatic morphological changes at 30 hours were reflected by the pancreatic histopathological scores (Figures 1(b)–1(e)). CQCQD treatment alone did not affect pancreas morphology (Figure S1A, in Supplementary Material available online at https://doi.org/10.1155/2017/5864945).

**Figure 2:**
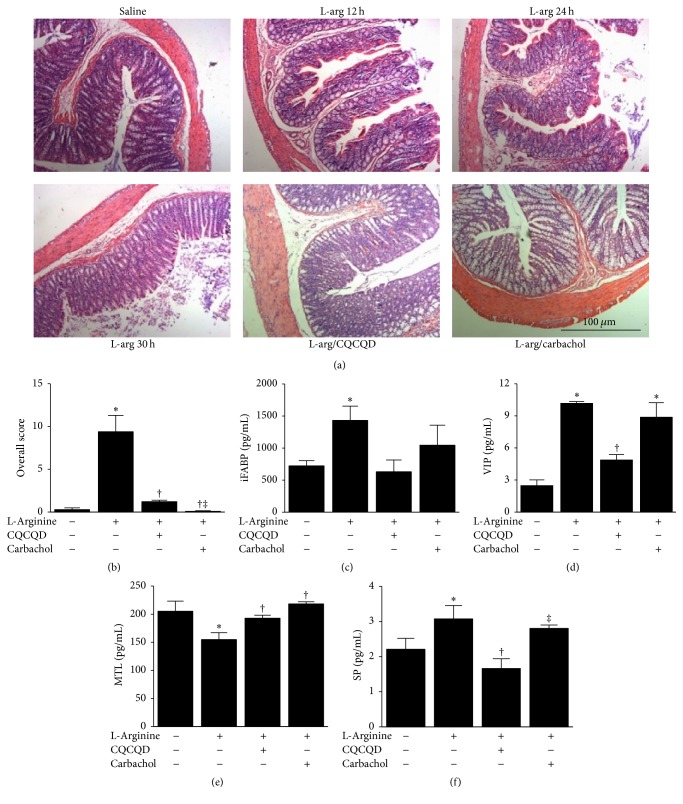
Effects of CQCQD and carbachol on colonic histopathology and serum intestinal motility parameters. (a) Representative H&E sections of upper colon (×200). (b) Overall histopathological score of colon. Indirect intestinal motility serum markers: (c) intestinal fatty acid binding protein (iFABP), (d) vasoactive intestinal peptide (VIP). (e) Motilin (MTL) and (f) substance P (SP). ^*∗*^*P* < 0.05 versus control group; ^†^*P* < 0.05 versus L-arginine group; ^‡^*P* < 0.05 versus CQCQD treated group. Values are means ± SEM of 5–11 animals per group.

Characteristic H&E colonic sections are demonstrated in Figure 2(a). Saline injections did not cause significant changes of colonic morphology. At 12 hours of L-arginine injection, there were scattered loss of villi and Crypt damage; at 24 hours, this damage became more apparent and was associated with significant inflammatory cell infiltration; at 30 hours, marked loss of villi and Crypt damage with diffused inflammatory cell infiltrations were observed. The histopathological changes of colon were demonstrated in Figure 2(b). L-Arginine also significantly affected intestinal motility markers iFABP, VIP, MTL, and SP. L-Arginine significantly increased serum iFABP (Figure 2(c)), VIP (Figure 2(d)), and SP (Figure 2(f)) levels and reduced MTL (Figure 2(e)) levels. CQCQD alone affected neither colonic histopathology (Figure S1B) nor intestinal motility parameters (Figure S1C).

**Figure 3:**
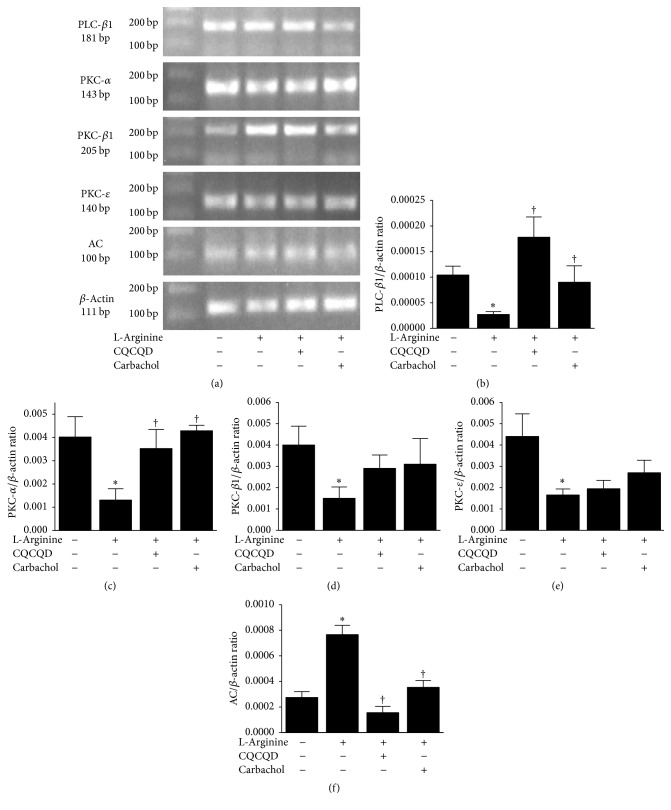
Effects of CQCQD and carbachol on mRNA expression of PLC-*β*1, PKC isoforms, and AC in colonic SMCs. (a) Representative images of agarose gel electrophoresis of targeted RT-PCR mRNAs. (b) PLC-*β*1 mRNA, (c) PKC-*α*, (d) PKC-*β*, (e) PKC-*ε*, and (f) AC. Relative expression of individual mRNA is calculated according to *β*-actin. ^*∗*^*P* < 0.05 versus control group; ^†^*P* < 0.05 versus L-arginine group. Values are means ± SEM of 5–11 animals per group.

**Figure 4:**
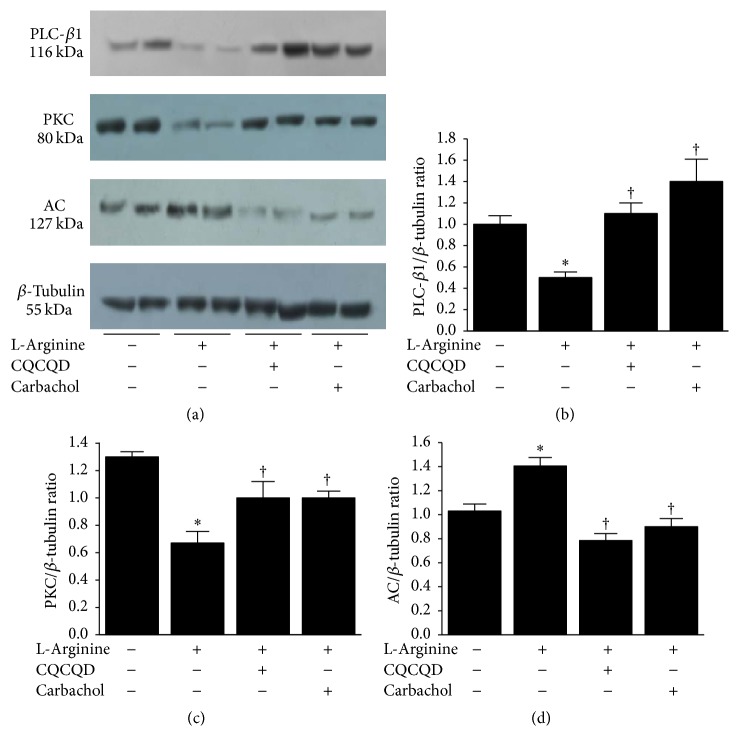
Effects of CQCQD and carbachol on protein expression of PLC, PKC, and AC in colonic SMCs. (a) Representative Western-blotting images of proteins. (b) PLC, (c) PKC, and (d) AC. Relative expression of individual mRNA is calculated according to *β*-tubulin. ^*∗*^*P* < 0.05 versus control group; ^†^*P* < 0.05 versus L-arginine group. Values are means ± SEM of 5–11 animals per group.

**Figure 5:**
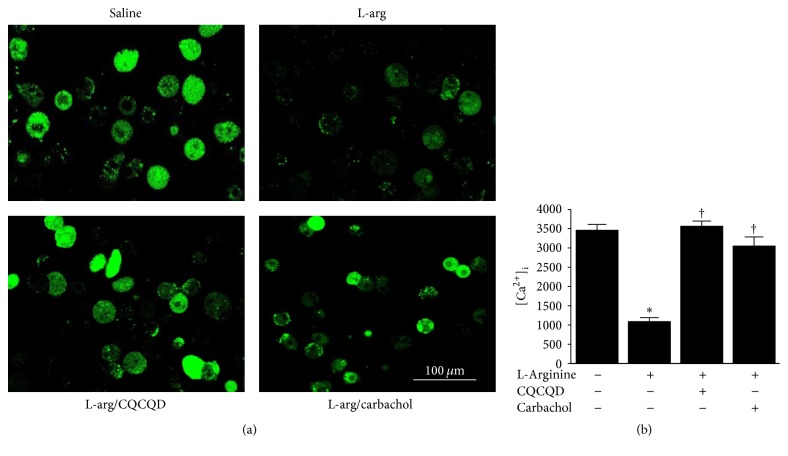
Effects of CQCQD and carbachol on [Ca^2+^]_i_ in colonic SMCs. (a) Representative confocal microscopic images of Fluo 3-AM stained SMCs (×600). (b) Fluo 3-AM fluorescence intensity (FI) for experimental groups. ^*∗*^*P* < 0.05 versus control group; ^†^*P* < 0.05 versus L-arginine group. Values are means ± SEM of 5–11 animals per group.

The images of isolated colonic SMCs are shown in Figure S2. The shape of SMCs appeared as oval, short spindle, or polygonal. After a-SMA and eosin staining, the morphological features of SMCs exhibited long spindle-shape. The nuclei and cytoplasm appeared as light blue and brown under the microscope, respectively. The cellular borders were unruffled and smoothly shaped and cytoplasm was homogeneously distributed. Illustrative images of agarose gel electrophoresis and Western-blotting analyses for mRNA and protein are shown in Figures 3(a) and 4(a). L-Arginine injections induced significant reduction of mRNA for PLC-*β*1 and PKC isoforms (*α*, *β*1, *ε*) when compared with saline controls (Figures 3(b)–3(e)), while the AC mRNA was significantly increased (Figure 3(f)). Further analyses confirmed that proteins PLC-*β*1 (Figure 4(b)) and PKC (Figure 4(c)) were reduced and AC (Figure 4(d)) was increased. Typical fluorescent images of Fluo 3-AM reflecting [Ca^2+^]_i_ in isolated colonic SMCs are displayed in Figure 5(a). L-Arginine caused reduction of [Ca^2+^]_i_ FI to less than 1/3 of the saline controls (Figure 5(b)).

### 3.2. CQCQD and Carbachol Alleviate Severity of Pancreatic and Colonic Histopathology and Improve Intestinal Motility

Both CQCQD and carbachol treatments reduced the overall histopathological score of the pancreas (mean score of 2.5 and 5.0 versus 6.9, both *P* < 0.05), with the effect of CQCQD significantly better than carbachol (*P* < 0.05; Figure 1(b)). Both CQCQD and carbachol treatments reduced oedema, neutrophil infiltration, and acinar necrosis, although CQCQD appeared to have greater effect than carbachol (Figures 1(c), 1(d), and 1(e)). Both CQCQD and carbachol protected the colonic damage shown by the reduction of overall colonic histopathology score (mean score of 1 and 0.1 versus 9.4, both *P* < 0.05; Figure 2(b)) and significantly reduced VIP and SP (all *P* < 0.05), with a trend towards decreasing iFABP (Figures 2(c)–2(f)). Both CQCQD and carbachol significantly elevated serum MTL levels (both *P* < 0.05).

### 3.3. CQCQD and Carbachol Regulate PKC-Mediated Ca^2+^ Release in Colonic SMCs

Both CQCQD and carbachol significantly increased PLC-*β*1 mRNA (Figure 3(b)) and PLC protein expression (Figure 4(b)) as well as PKC-*α* (Figure 3(c)) and PKC protein expression (Figure 4(c)). Neither CQCQD nor carbachol significantly altered the mRNA of other PLC isoforms (Figures 3(d) and 3(e)). Both CQCQD and carbachol reduced AC mRNA (Figure 3(f)) and protein expression (Figure 4(d)) to similar levels compared to controls. Both CQCQD and carbachol significantly increased the L-arginine-induced reduction of [Ca^2+^]_i_, FI, the levels of which were normalised when compared with controls (Figure 5(b)).

## 4. Discussion

This is first study to investigate the effects of CQCQD and carbachol on PLC-mediated Ca^2+^ signalling in colonic SMCs in the setting of experimental AP. L-Arginine caused necrotising AP, colonic damage, altered indirect intestinal motility serum markers, and deranged PLC-mediated Ca^2+^ signalling in colonic SMCs. Both CQCQD and carbachol decreased these effects, and CQCQD was more effective than carbachol. This experimental evidence suggests that these prokinetic agents can improve gut dysfunction in severe AP. Furthermore, the evidence suggests that they act through PKC-mediated Ca^2+^ signalling pathways.

Not only does gut injury occur as a result of AP [9, 10, 17], but also it contributes to the severity of AP [9]. It has recently been shown that gut-associated lymphoid tissue has increased pancreatic protease, inflammatory cells, cytokines, and damage-associated molecular pattern molecules, which are released into circulation exacerbate systemic inflammation [19, 20]. Traditional Chinese Medicine classifies intestine as one of the six FU organs. Based on the theory that “hollow viscera function well when unobstructed,” the treatment formulas for AP tend to contain purgatives such as rhubarb and mirabilite. In West China Hospital and elsewhere in China, there has been the important development of a “gut-centred” therapeutic strategy in the early management of AP. CQCQD and its analogs all contain the aforementioned purgatives and have been used for treating AP for more than three decades in West China Hospital using an integrated Traditional Chinese and Western Medicine approach [41, 42]. From clinical studies to date this integrated approach has demonstrated an overall reduction in adverse clinical outcomes, including a reduction in gut dysmotility [33–35], respiratory failure [43], and proinflammatory cytokines [44]. While encouraging, it is clear that the priority is now to conduct studies for a higher level of evidence regarding the efficacy of Chinese herbal medicine to reduce gut dysfunction in AP.

In this study, it was shown that L-arginine-induced colonic damage with the increase of iFABP, VIP, and SP levels as well as decrease of MTL levels. iFABP [17] and VIP [15, 35] are widely used indirect parameters for intestinal motility assessment. SP is known as a neurotransmitter, but it has been shown that it also has a direct effect on intestinal SMCs by promoting mechanical contraction and bursts of spike potentials [45]. The increased serum SP levels can be a reflection of systemic inflammation but also serves as a feedback mechanism for impaired intestinal motility. On the other hand, MTL acts on motilin receptors to initiate phase III of gastric migrating myoelectric complexes to improve intestinal motility [46]. It has been shown in a clinical study that serum MTL levels were significantly decreased in AP patients when compared with heathy volunteers and this correlated with AP severity [15]. Collectively, these findings support the use of serum MTL and SP as indirect measures for intestinal motility. CQCQD had a significant effect on MTL and SP levels, returning them towards normal range. The result is consistent with what has been reported with the CQCQD (and its derivatives) treatment of patients with AP [34, 35, 41, 42]. Despite having only minor effect on serum SP, carbachol significantly increased serum MTL levels, indicating a potential role in the promotion of gut motility in experimental AP.

Early studies have shown that total and segmental colonic transit time was delayed in human AP and associated with disease severity [15]. In experimental necrotising AP, the colonic motility was impaired, evidenced by significantly reduced amplitude of peristaltic waves and prolonged peristaltic contraction intervals in isolated colonic segment from these mice [47]. In this study prokinetic agents (a serotonin receptor agonist) improved motility both in vitro and in vivo [47]. These data are consistent with our strategy to improve intestinal motility as part of the treatment of AP. Multiple different mechanisms could be exploited to promote intestinal motility including the use of intestinal peptides [21], serotonin [47], ghrelin, and motilin via their respective receptors [46]. The muscarinic cholinergic receptor subtypes such M2 and M3 also hold promise. In the muscarinic M3 receptor signalling pathway, signals through PLC-*β* activation promote generating diacylglycerol which is responsible for PKC activation [21–23]. Our data show that L-arginine dramatically reduced both PLC-*β*1 mRNA and PLC protein. The downstream PKC isoform (*α*, *β*, and *ε*) mRNA and PKC protein were also greatly reduced by L-arginine. Both CQCQD and carbachol restored PLC-*β*1 and PKC-*α* mRNAs and proteins but did not significantly affect other PKC isoforms isoform (*β*, *ε*). The activation of M2 receptor leads to inhibition of cyclic adenosine monophosphate which in turn inhibits the effects of relaxant reagents on M3 receptor in SMCs, and both synergistically improve contractibility [48]. The observation of increased AC mRNA and protein by L-arginine was in agreement with this mechanism, and both CQCQD and carbachol significantly decreased AC expression similar to saline controls. Increase of PLC-*β*1 and PKC-*α* as well as decrease of AC expression eventually resulted in elevation of [Ca^2+^]_i_ in colonic SMCs and thus improved intestinal motility and pancreatic histopathology. These findings indicate that impairment of the PKC-mediated signalling pathway in colonic SMCs played a role in the pathogenesis of intestinal dysmotility in experimental AP. The decreased pancreatic injury would also contribute to the improvement of intestinal motility as CQCQD significantly more reduced pancreatic injury than carbachol and thus better improved overall intestinal motility parameters, albeit that carbachol had equivalent or better effects on colonic histopathology and regulating PLC signalling pathways in colonic SMCs.

There were several limitations in this study. The L-arginine-induced AP has marginal clinical relevance, and this study should be repeated using other AP models included by bile acids, fatty acid ethyl esters [49], and hyperlipidaemia [50]. Furthermore, it is noteworthy that L-arginine at high doses directly causes intestinal injury and dysmotility by releasing neurotransmitter (i.e., nitric oxide) [51]. Therefore, future studies using pancreatic toxins that have no direct effects on intestine would be ideal for this type of research. In this study, we did not directly measure gut motility, but it is noted that a CQCQD derivate has been shown to reduce intestinal mucosal injury in a sodium taurocholate-induced AP model in rats and improves intestinal propulsion index in patients [35].

## 5. Conclusions 

Both CQCQD and carbachol appear to improve intestinal motility in L-arginine-induced experimental necrotising AP, as reflected by changes of colonic histopathology and indirect intestinal motility parameters. The protective effect on intestinal motility may result from improved pancreatic injury and appears to be mediated by PKC-medicated Ca^2+^ release in colonic SMCs, supporting the view that aberrant PKC-mediated signalling pathways in intestinal cells have a role in the pathogenesis of intestinal dysmotility. These results provide support for clinical trials of these prokinetic treatments in AP.

## Supplementary Material

CQCQD treatment alone did not effect pancreatic and colonic morphology as well as intestinal motility parameters.

## Figures and Tables

**Table 1:** Probe, primer, and product (bp) in RT-PCR.

Gene target	Probe	Upstream primer	Downstream primer	Product (bp)
PLC-*β*1	CCTCCTCATGAACTCTGGCTTC	CCAGACAGTGGATCTAGCTATG	CTGACCTGAAATAATCTTAACAG	**181**
PKC-*α*	CCTCCTCATGAACTCTGGCTTC	GCTGAGGCAGAAGAACGTGCAT	CAAAACAGCAAACTTGGCACTG	**143**
PKC-*β*1	GTCTTGGTTGTCACCCCATCCC	GAAACTTGACAACGTGATGCTG	CAACATTTCATACAGCAGGACT	**205**
PKC-*ε*	GAATCCCTTCCTTGCACATCCC	CTACAGGGATTTGAAACTGGAC	GAGCTATGTAGTCAGGAGTCCC	**140**
AC	TGCTATCCATCCGACTGGCCAC	CAGGCCCCAGTACGACATCTGG	GTGGACTTCCTCAGTCACCTGA	**100**

*β*-actin	TCACTGTCCACCTTCCAGCAGA	GAAGATCAAGATCATTGCTCCT	TACTCCTGCTTGCTGATCCACA	**111**
