# Supplementary material for: Chai-Qin-Cheng-Qi Decoction and Carbachol Improve Intestinal Motility by Regulating Protein Kinase C-Mediated Ca2+ Release in Colonic Smooth Muscle Cells in Rats with Acute Necrotising Pancreatitis
Source: Evid Based Complement Alternat Med. 2017 Apr 26;2017:5864945. doi: 10.1155/2017/5864945 (PMC5424168; doi:10.1155/2017/5864945)

Supplementary Materials include one Figure.

Figure S1: Morphological observation of isolated colonic SMCs. (A) Light microscopic appearance. (B) Stained with α-SMA.

Figure S1

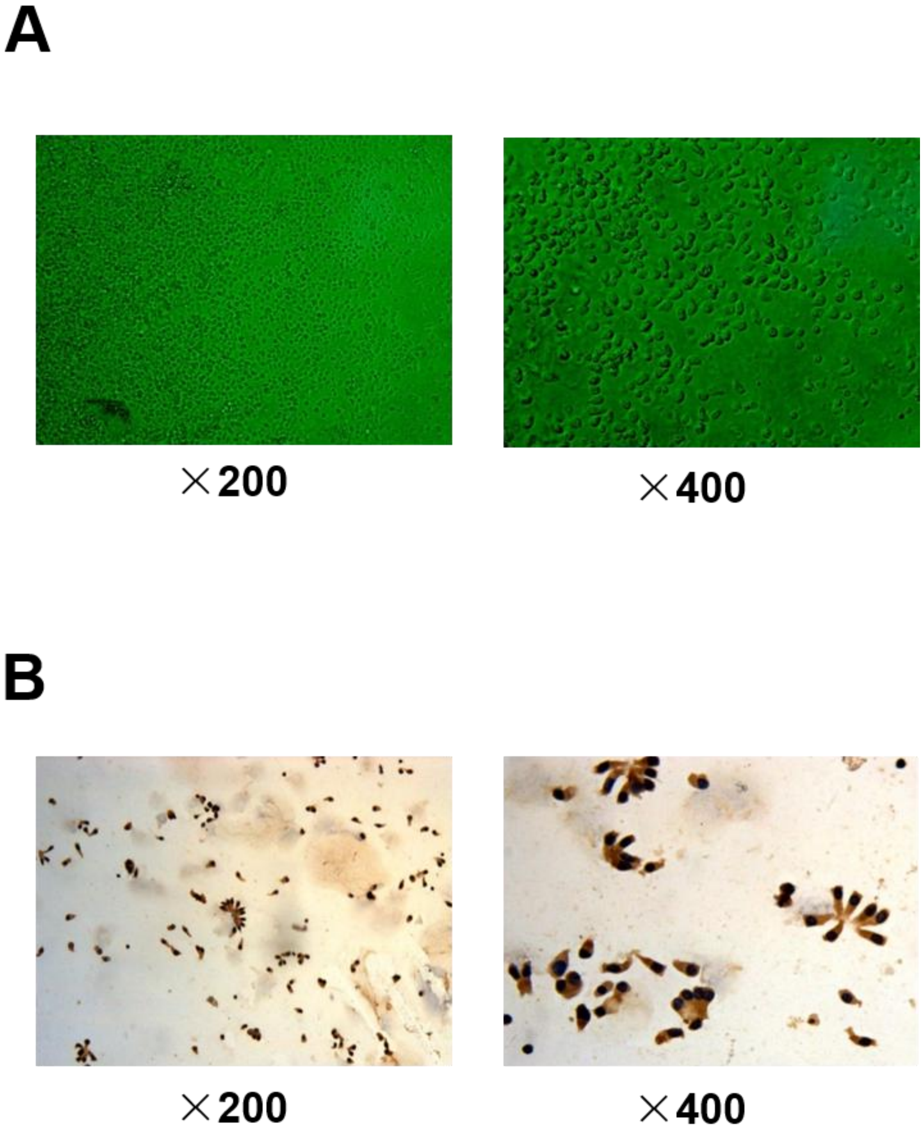

Supplement: Supplementary file 1 — CQCQD treatment alone did not effect pancreatic and colonic morphology as well as intestinal motility parameters. [file 5864945.f1.pdf]
